# Vasodilator Compounds Derived from Plants and Their Mechanisms of Action

**DOI:** 10.3390/molecules18055814

**Published:** 2013-05-17

**Authors:** Francisco J. Luna-Vázquez, César Ibarra-Alvarado, Alejandra Rojas-Molina, Isela Rojas-Molina, Miguel Ángel Zavala-Sánchez

**Affiliations:** 1 Doctorado en Ciencias Biológicas y de la Salud, Universidad Autónoma Metropolitana, Unidad Xochimilco, México, D.F. 04960, Mexico; 2 Laboratorio de Investigación Química y Farmacológica de Productos Naturales, Facultad de Química, Universidad Autónoma de Querétaro, Querétaro, Qro. 76010, Mexico; 3 Departamento de Sistemas Biológicos, Universidad Autónoma Metropolitana, Unidad Xochimilco, México, D.F. A.P. 23-181, Mexico

**Keywords:** vasodilator compounds, vascular endothelium, arterial smooth muscle, NO/cGMP pathway, PGI_2_/cAMP pathway, potassium channel activators, calcium channel blockers, phosphodiesterases inhibitors, PKC inhibitors

## Abstract

The present paper reviews vasodilator compounds isolated from plants that were reported in the past 22 years (1990 to 2012) and the different mechanisms of action involved in their vasodilator effects. The search for reports was conducted in a comprehensive manner, intending to encompass those metabolites with a vasodilator effect whose mechanism of action involved both vascular endothelium and arterial smooth muscle. The results obtained from our bibliographic search showed that over half of the isolated compounds have a mechanism of action involving the endothelium. Most of these bioactive metabolites cause vasodilation either by activating the nitric oxide/cGMP pathway or by blocking voltage-dependent calcium channels. Moreover, it was found that many compounds induced vasodilation by more than one mechanism. This review confirms that secondary metabolites, which include a significant group of compounds with extensive chemical diversity, are a valuable source of new pharmaceuticals useful for the treatment and prevention of cardiovascular diseases.

## 1. Introduction

According to the World Health Organization, cardiovascular diseases are the leading cause of death worldwide. Among these, arterial hypertension has a high prevalence and is associated with other conditions, such as myocardial infarction and stroke [1]. Although there are more than 200 drugs that lower blood pressure, less than a third of the hypertension cases are successfully treated due to their low efficacy, detrimental side effects and lack of cardiovascular risk reduction [2]. In addition, the etiology of hypertension has been associated with vascular endothelial dysfunction, which is characterized by an uncoupling between the release of endothelial factors such as nitric oxide (NO), prostacyclin (PGI_2_) and endothelium-derived hyperpolarization (EDH), as well as effects on endothelium-dependent contractile mechanisms, and the associated change in vascular smooth muscle tone [3].

Some studies have suggested that changes in the bioavailability of endothelium-derived NO may be responsible for endothelial dysfunction and the related altered blood pressure and myocardial infarction [4,5,6,7,8,9,10]. Such altered NO levels can be due to dysfunction of soluble guanylate cyclase protein (sGC), with changes in the levels of this protein likely related to the pathophysiology of pulmonary hypertension and hypoxia [11,12]. With regard to vascular smooth muscle relaxation, various cardiovascular diseases, such as coronary vasospasm [13,14], cardiac ischemia [15] and hypertension [16] have also been associated with altered expression and activation of various potassium channels. Based on the above evidence, we are currently seeking new therapeutic strategies for preventing and treating these conditions that also have relaxing effects on vascular smooth muscle. 

In this context, plants are a major source of new biologically active compounds, and the ethnomedical knowledge of traditional medicine from around the world is a useful starting point for determining their efficacy. In addition, due to the multifactorial nature of cardiovascular disease such as hypertension, knowledge of the mechanisms of action of each of the compounds proposed for use in the treatment for this disease is a crucial element for planning and developing different therapeutic strategies. Therefore, the present work reviews the previously reported vasodilator compounds isolated from plants and the different mechanisms of action involved in their vasodilator effects.

## 2. Search Strategy

The literature review focused on the past 22 years (1990 to 2012), taking into account studies on the vasodilating activity of plant-based treatments and the compounds derived from them. We reviewed more than 450 abstracts on this topic. The search was focused on those metabolites with a vasodilator effect whose mechanism of action involved the vascular endothelium and the arterial smooth muscle vasorelaxation pathways; we did not consider the antioxidant activity or reactive oxygen species scavenging.

## 3. Types of Compounds with Vasodilator Effects

**Figure 1:**
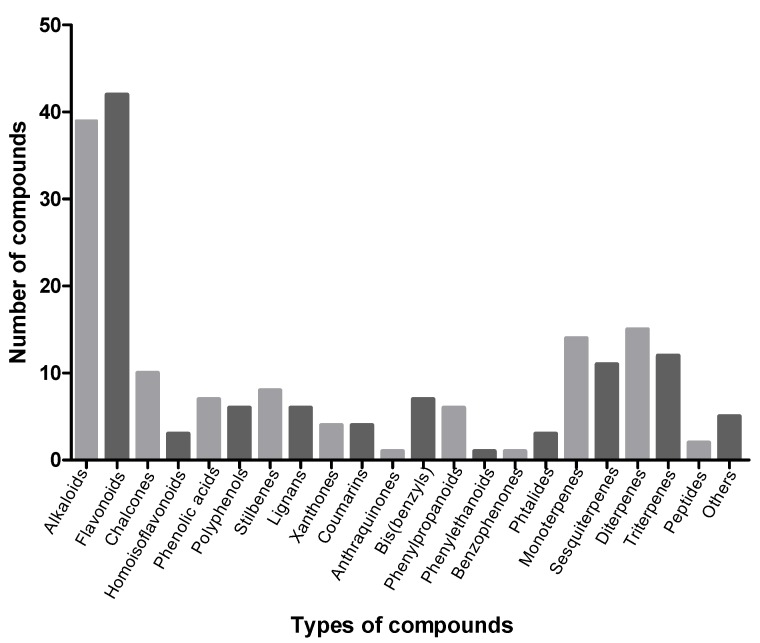
Classification of vasodilator compounds obtained from plants according to their chemical nature.

We identified 207 vasodilator metabolites together with their possible mechanism(s) of action. First, these compounds were classified according to their chemical nature. It is clear that most compounds with vasodilator activity are alkaloids, flavonoids, or terpenoids (Figure 1). The classification of these compounds offers an overview of the types of compounds that present significant vasodilator activity and of the structural diversity exhibited by these bioactive compounds.

Some of these compounds have been studied on multiple occasions, and various mechanisms of action have been proposed to explain their vasodilatory activities. These compounds include the flavonoids naringenin [17,18,19], dioclein [20,21,22,23], quercetin [24,25,26,27,28] and (−)-epigallocatechin-3-gallate [29,30,31]; the polyphenols piceatannol [32,33] and resveratrol [34,35,36]; the sesquiterpene polygodial [37,38,39]; the monoterpene rotundifolone [40,41,42] and the alkaloid rutaecarpine [43,44,45,46].

In other cases, mixtures of various compounds obtained from plants or the products generated from them were studied; examples include polyphenols in red wine [47,48], saponins from ginseng [49], proanthocyanidins from persimmon leaf tea [50] and green tea [48,51], as well as the xanthones obtained from *Halenia elliptica* [52]. In 34 plants, two or more vasodilator compounds were identified, which in some cases had different mechanisms of action. Examples of this are the chalcones isolated from *Angelica keiskei* [53], the alkaloids obtained from *Peganum harmala* [54], the glycosides identified in *Melaleuca quinquenervia* [55] and the macrocyclic bis(bibenzyls) from liverworts [56]. In these examples, the fundamental difference between the mechanisms of action proposed for the isolated compounds is based on their dependence or independence on the endothelium, the involvement of the NO/cGMP pathway and the blockage of voltage-dependent Ca^2+^ channels.

## 4. Proposed Mechanisms of Action

**Figure 2:**
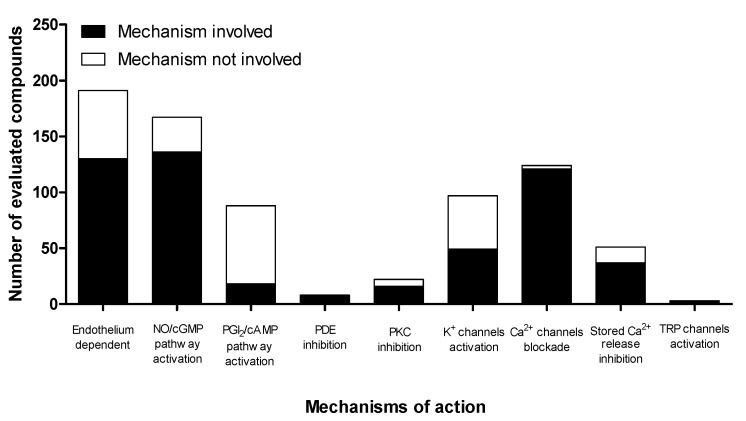
Classification of compounds obtained from plants according to the main mechanism(s) of action involved in their vasodilator effect.

Different mechanisms of action were proposed to explain the vasodilator effect of the 207 compounds derived from plants (Figure 2).

Analysis of the mechanisms of action of these compounds revealed that, on the one hand, the vasodilator effect of a significant number of compounds (40%) involves two or more mechanisms (Table 1). On the other hand, as shown in Figure 2, over half of the tested compounds have a mechanism of action that requires the participation of the endothelium, at least in part. Therefore, endothelium-derived factors play a key role in the mechanisms of action of these vasodilators. The mechanisms of action most frequently assessed in the vasodilator effects of the plant compounds were activation of the NO/cGMP pathway, blockade of Ca^2+^ channels, and activation of K^+^ channels.

## 5. Participation of the Endothelium in the Mechanism of Action

The vascular endothelium synthesizes and releases a broad spectrum of vasoactive substances and plays a fundamental role in the regulation and maintenance of cardiovascular homeostasis [57]. Among the main endothelial-derived factors that relax arterial smooth muscle are NO [58,59], PGI_2_ [59,60] and the EDH mechanism, which is associated with calcium-activated potassium channel activation [59,61]. Approximately one third of the compounds analyzed utilized both endothelium-dependent and endothelium-independent mechanisms (Table 1). Moreover, among the compounds that produce their vasodilator effect by an endothelium-dependent mechanism, a high percentage (98.4%) involved the NO/cGMP pathway, whereas the PGI_2_/cAMP pathway was involved in the mechanism used by a low percentage (23%) of the vasodilating compounds (Table 1). Among the 130 compounds whose mechanism of action was endothelium-dependent, assays for evaluating the participation of endothelial muscarinic receptors were performed in only 18. Four of these compounds involved the participation of this kind of receptors: diosgenin [62], reticuline [63], rotundifolone [40] and ursolic acid [64].

**Table 1:** Mechanisms of action proposed for vasodilator compounds obtained from plants.

	Compound	Type of artery/vein	EC_50_	Endothelium	NO/cGMP	PGI_2_/cAMP	PDE	PKC	K^+^ Ch	Ca^2+^_ext_ /Ca^2+^_int_	Ref.
1	Allicin	rat pulmonary	0.8 µg/mL ^1^	d	+	x					[65]
2	Allyl isothiocyanate	rat cerebral	164 µM ^2^	d	x	x			+IK_Ca_,	+TRPA1/	[66]
									+SK_Ca_,		
									+K_IR_		
3	Alpha-terpineol	rat mesenteric	NR		+						[67]
4	Alpha-zearalanol	rat aorta	NR	d/i	+				+BK_Ca_,	-VOCC/	[68]
									+K_ATP_		
5	Alpinetin	rat mesenteric	27.5 µM ^1^	d/i	+	x		-		-VOCC/ -IP_3_R, -RyRs	[69]
6	Alstonisine	rat aorta	NR	d/i	+				x	-VOCC,-	[70]
										ROCC/	
7	Amentoflavone	rat aorta	NR	d	+	x			+	-VOCC/	[71]
8	Angelic ester of 2-β-hydroxy-8α-*H*-7(11)-eremophilene-12,8-olide	rat mesenteric	4.74 ± 0.1 ^§,2^							-VOCC_L_/	[72]
		rat aorta	5.43 ± 0.06 ^§,2^								
9	Angelic ester of 2-β-hydroxy-8β-*H*-7(11)-eremophilene-12,8-olide	rat mesenteric	4.11 ± 0.02 ^§,2^		x	x				-VOCC_L_/x	[72]
		rat aorta	4.92 ± 0.09 ^§,2^								
10	Apigenin	rat aorta	3.7 ± 0.5 µM ^1^	d/i	+ x	x		x	+IK_Ca_, +SK_Ca_	-VOCC, -ROCC/x +TRPV4/	[73]
		rat aorta	63 µM ^5^	i							[74]
		rat mesenteric	NR	d							[75]
11	Apocynin	rat aorta	780 ± 80 µM ^1^	d/i	+	x			+K_ATP_	-VOCC,- ROCC/ -IP_3_R	[76]
12	Astragaloside IV	rat aorta	NR	d/i	+	+				-VOCC,- ROCC/ -IP_3_R	[77]
13	Backebergine	rat aorta	NR	d/i	+				x	-VOCC,- ROCC/	[78]
14	Baicalin	rat mesenteric	NR	i	+	+		-	+BK_Ca_	-VOCC/	[79]
15	4-Benzoyl-2-C-β-gluco-pyranosyl-3,5-dihydroxy-6-methylphenyl β-d-glucopyranoside	rat aorta	NR	d	+						[55]
16	Berberine	rat mesenteric	1.48 ± 0.16 µM ^1^	d/i	+	x		x	+BK_Ca,_ +K_v_,	x/-RyRs	[80]
									+K_IR_		
17	Betulinic acid	rat aorta	1.67 µM ^1^	d	+	x					[81]
18	Bilobalide	rat aorta	NR		+				x	-VOCC/	[82]
19	Biochanin A	rat aorta	NR	i					+BK_Ca_, +K_ATP_	-VOCC,- ROCC/-	[83]
20	Brazilin	rat aorta	183 ± 30 µM ^1^	d	+						[84]
		rat aorta		i	x						[85]
		rat mesenteric		i	x						[85]
21	(−)-Borneol	rat aorta	4.63 ± 0.15 ^§,1^	i					+BK_Ca_, +K_v_,	-VOCC_L_/-	[86]
									+K_ATP_		
22	Butein	rat aorta	7.4 ± 1.6 µM ^1^	d	+	x	-		x		[87]
23	Butylidenephthalide	rat aorta	4.20 ± 0.07 ^§,3^	d/i	+	x		-	x	-VOCC_L_, -ROCC/-	[88]
24	Cadamine	rat aorta	NR	d/i	+				x	-VOCC,- ROCC/	[89]
25	Caffeic acid	rat aorta	400 µM ^1^	d/i	+				x		[90]
26	Caffeic acid phenethyl ester	porcine coronary	4.99 ± 0.17 ^§,1^	d/i	+					-VOCC/	[91]
		rat aorta	5.15 ± 0.0 ^§,4^	d	+	x					[92]
27	Calycosin	rat aorta	4.46 ± 0.13 ^§,3^	i	x	x				-VOCC/x	[93]
28	Capsaicin	rat mesenteric	NR		x						[94]
29	Cardamonin	rat mesenteric rat tail	9.3 µM ^1^ 4.63 ± 0.01 ^§,1^	d/i	+	x		-	+BK_Ca_	-VOCC/- IP_3_R,-RyRs -VOCC/	[69] [95]
30	Carvacrol	rat aorta rat cerebral	145.4 ± 6.07 µM ^1^	id	x	x		-	+SK_Ca_, +K_IR_, +IK_Ca_	-VOCC/-IP_3_R +TRPV3/	[96] [97]
			78.8 ± 11.9 µM ^2^								
			4.1 µM								
31	Cassiarin A	rat mesenteric	6.4 ± 0.8 µM ^1^	d/i	+	x			+BK_Ca_		[98]
32	Cathafoline	rat aorta	NR	d/i	+				x	-ROCC/	[70]
33	Centaureidin	rat orta	16.7 ± 1.9 µM ^5^	i							[99]
34	Chrysin	rat orta	16 ± 4 µM ^1^	d	+						[100,101]
35	Chrysin glucoside	rat aorta	52 µM ^5^	d/i	+						[102]
36	Cinnamaldehyde	rat aorta	NR	d/i	+	x			x	-VOCC/	[103]
37	Ethyl cinnamate	rat aorta	380 ± 40 µM ^1^	d/i	+	+				-VOCC/	[104]
38	1,8-Cineole	rat aorta	663.2 ± 63.8 µg/mL ^1^	d	+	x			x		[105]
39	(+)-*cis*-4'-*O*-Acetyl-3'- *O*-angeloylkhellactone	rat aorta	NR	d/i	+	x			x	-VOCC/	[106]
40	Citral	rat aorta	NR	d/i	+	x				-ROCC/-	[102]
41	Citronellol	rat mesenteric	0.71 ± 0.11 ^§,1^	i					x	-VOCC/- IP_3_R, -RyRs	[107]
42	Coptisine	rat aorta	4.49 ± 0.48 ^§,5^	d/i	+	+			+K_V_	-VOCC,- ROCC/-	[108]
43	Cornuside	rat aorta	NR	d	+	x			x		[109]
44	Cryptotanshinone	rat coronary	2.65 ± 0.15 µg/mL 6	i	x	x			x	-VOCC_L_/	[110]
45	Curine	rat mesentericrat aorta	4.8 ± 1.9 µM ^5^ 7.6 ± 1.6 µM ^1^	i						-VOCC/- - VOCC_L_/-	[111] [112]
46	Curcumin	porcine coronary	6.28 ± 0.28 µM ^4^	d	+	x					[113]
47	Cyclosquamosin B	rat aorta	NR	i						-VOCC/	[114]
48	Daidzein	rat basilar	20 ± 7 µM ^3^ 7.4 ± 1.9 µM^6^	ii	x	x			+ +BK_Ca_, +K_ATP_	-VOCC/	[115] [116]
49	Daidzin	rat basilar	140 ± 21 µM ^3^	i	x	x			+K_ATP_	-VOCC/	[115]
50	Danshensu	rat coronary	71.5 ± 11 µg/mL ^6^	i					+	-VOCC_L_/	[117]
51	Dehydroevodiamine	rat mesenteric	NR	d/i	+	x			+	-VOCC/	[118]
52	Demethylpiperitol	rat aorta	NR	d	+						[119]
53	Denudatin B	rat aorta	21.2 µg/mL ^2^	i	↑cGMP	x				-VOCC,- ROCC/x	[120]
54	14-Deoxy-andrographolide	rat aorta	NR	d /i	+	x			x	-VOCC, -ROCC/	[121]
55	Dictamnine	rat aorta	15 µM ^2^	i					x	-VOCC,- ROCC/	[122]
56	Dihydrotanshinone	rat coronary	10.39 ± 1.69 µM ^6^	i	x	x			x	-VOCC_L_/	[123]
57	3,7-Dihydroxy-2,4-dimethoxyphenanthrene	rat aorta	NR	d/i	+						[124]
58	Dioclein	rat aorta	1.3 ± 3.1 µM ^1^	d	+	x x	-	-	+K_Ca_, +K_V_	-VOCC/-IP_3_R	[20]
		rat aorta	350 ± 80 µM ^5^	i							[21]
		rat mesenteric	0.3 ± 0.06 µM ^1^	d/i							[22]
		human saphenous	7.3 ± 3.1 µM ^1^	i							[23]
59	Diosgenin	rat mesenteric	330 ± 120 µM ^1^	d	+	+			+BK_Ca_		[62]
60	Echinacoside	rat aorta	NR	d	+	x					[125]
61	Ellagic acid	rat aorta	5.60 ± 0.03 ^§,1^	d/i	+	x			x	-VOCC_L_/	[126]
62	Emodin	rat aorta	NR	i	↑cGMP						[127]
63	Ent-18-hydroxy-trachyloban-3-one	rat aorta	5.7 ± 0.01 ^§,2^		x					-VOCC_L_/	[128]
64	Ent-8(14), 15-pimaradien-3β-ol	rat aorta	4.8 ± 0.1 ^§,1^	d/i	+	x			x	-VOCC/x	[129]
65	Epicatechin	rat aorta	4.72 ± 0.07 ^§,1^	d	+						[130]
66	7-Epiclusianone	rat aorta	NR	d	+	x					[131]
67	(−)-Epigallocatechin-3-gallate	rat aorta	191.8 ± 13 µM ^5^	i			-		x +BK_Ca_		[29]
		bovine ophtalmic	6.21 ± 0.06 §,6	d	+						[31]
		rat aorta	4.76 ± 0.07 ^§,1^	d	+						[130]
68	Equol (daidzein metabolite)	rat aorta	NR	d	+						[132]
69	Eriodictyol	rat aorta	61.1 ± 2 µM ^5^	i				x		-VOCC/	[133]
70	Erythrodiol	rat aorta	3.38 ± 1.27 µM ^1^	d	+	x					[134]
71	Eudesmin	rat aorta	10.69 ± 0.77 µg/mL ^1^	d	+	+					[135]
72	Eugenol	rat aorta				x			x	- VOCC,- ROCC/x - VOCC,- ROCC/	[136]
		rat aorta	1200 µM ^1^	d/i	+						[137]
		rat mesenteric		d/i	x						[138]
73	Euxanthone	rat aorta	32.5 ± 2.5 µM ^5^	i	x	x		-	x	-VOCC,- ROCC/ -IP_3_R	[139]
74	Evocarpine	rat aorta	9.8 µM ^2^							-VOCC/	[140]
75	Evodiamine	rat mesenteric	NR	d/i					x	-ROCC/x	[141]
76	Ferulic acid	rat aorta	NR	i	x					x/	[142]
77	Floranol	rat mesenteric	19.9 ± 2.4 µM ^1^	d/i	+	x			x	-VOCC/	[143]
		rat aorta		i	x						[144]
78	Formononetin	rat aorta	NR	d/ i	+				+	-VOCC/	[145]
79	Forsythide	rat aorta	NR	i				x		-ROCC/	[146]
80	Fraxinellone	rat aorta	25 µM ^2^							-VOCC/	[122]
81	Galangin	rat aorta	NR	d/i	+	x				-VOCC/	[147]
82	Geissoschizine methyl ether	rat aorta	0.744 µM ^5^	d/i	+					-VOCC/	[148]
83	Genistein	rabbit coronary	NR	i	x	x			x	-VOCC_L_/	[149]
		human umbilical								-VOCC/-	[150]
84	Gigantol	rat aorta	NR	d/i	+						[124]
85	Ginsenoside Rg3	rat aorta	NR	d	+				+		[151]
86	Gomisin A	rat aorta	NR	d/i	+						[152]
87	Gymnopusin	rat aorta	63 µM ^5^	i	x				+BK_Ca_, +K_ATP_	-VOCC_L_/	[153]
88	Harmaline	rat aorta	32.8 ± 1.17 µM ^2^	d/i	+	+	-			-VOCC/	[154]
89	Harman	rat aorta	9 µM ^1^	d/i	+	x			x	-VOCC_L_, -ROCC/	[155]
90	Harmine	rat aorta	3.7 ± 1.2 µM ^5^	i	x	x	-			-VOCC/	[154]
91	Hematoxylin	rat aorta	NR	d	+						[156]
92	Hesperetin	rat aorta	62.8 ± 5.0 µM ^5^	i	x		-		x	-VOCC,- ROCC/	[157]
93	Hirsutine	rat aorta	10.6 µM ^5^	i						-VOCC/	[148]
94	4-Hydroxybenzoic acid	rat aorta	1780 µM ^1^	d	+				x		[90]
95	4-Hydroxyderricin	rat aorta	NR	d/i	+					-VOCC/	[53]
96	1-Hydroxy-2,3,5-trimethoxyxanthone	rat coronary	1.67 ± 0.27 µM ^6^	d	+	x		-	x	-VOCC_L_/x	[130]
97	Hypogallic acid	rat aorta	620 µM ^1^	d/ i	+				+K_ATP_		[90]
98	Icariin	rat aorta	NR		+						[158]
		canine coronary		d	+	x			x		[159]
99	Imperatorin	rat mesenteric mouse aorta		i					+BK_Ca_	-VOCC,- ROCC/-	[160]
			12.2 ± 2.4 µM ^1^	d	+						[161]
100	Isoliquiritigenin	rat aorta	7.4 ± 1.6 µM ^1^	i	↑cGMP	x			x		[162]
101	Isoplagiochin B	rat aorta	NR	i					+	-ROCC/	[56]
102	Isoplagiochin D	rat aorta	NR	i					x	-VOCC,- ROCC/	[56]
103	Isopropyl 3-(3,4-dihydroxyphenyl) -2-hydroxypropanoate	rat mesenteric	7.41 ± 0.08 ^§,5^	i					+BK_Ca_	-VOCC,- ROCC/-	[123]
104	Isorhamnetin	rat mesenteric	5.89 ± 0.11 ^§,5^	i	x	x					[163]
105	Isorhynchophylline	rat aorta	20–30 µM ^2^	i	x					-VOCC_L_/- IP_3_R	[164]
106	Iso-S-petasin	rat aorta	NR	i						-VOCC_L_/	[165]
107	Isotirumalin	rat aorta	4.84 ± 0.24 ^ǂ,1^	d	+						[166]
108	Jatrophone	rat aorta	11.0 µM ^5^	d/i					+	-VOCC/-	[167]
		rat portal vein	13.54 µM 5					-			[168]
109	Kaempferol	rat aorta rat aorta rat mesenteric porcine coronary rat aorta	580 µM ^1^ 4.81 ± 0.13 ^§,5^ 5.66 ± 0.06 ^§,5^	d/i d/i d	+ +						[90] [163] [163] [169] [170]
110	Kaurenoic acid	rat aorta	NR	d/i	+	x			+BK_Ca_, +K_V_	-VOCC/x	[171]
111	Keayanidine B	rat aorta	23.3 ± 1.3 µM ^1^		+						[172]
112	Keayanine	rat aorta	27.5 ± 2.4 µM ^1^		+						[172]
113	Kolaviron	rat mesenteric	NR	i					+BK_Ca_, +K_V_	-VOCC_L_/ - IP_3_R	[173]
114	Labdane-302	rat mesenteric	5.4 ± 1.4 µM ^1^	d/i	+	+				-VOCC_L_/	[174]
115	Labd-8 (17)-en-15-oic acid	rat aorta	313.6 µg/mL ^2^	i	x						[175]
116	Lectin (of *Pisum arvense*)	rat aorta	58.38 ± 1.87 µg/mL ^1^	d	+	x			x		[176]
117	Leonurine	rat aorta	86.4 ± 10.4 µM ^1^							- VOCC_L_/-	[177]
118	Leucocyanidol	rat aorta	2.75 ± 0.15 ^§,5^	d/i	+						[178]
119	Ligustilide	rat mesenteric	3.98 ^§,2^	i	x				x	-VOCC,- ROCC/ -RyR	[179]
		rat aorta	4.39 ± 0.11 ^§,1^	i	x	x			x		[180]
120	(−)-limacine	rat aorta	NR	d	+						[78]
121	Luteolin	rat aorta	NR	i	x				+K_IR_, +K_V_	-VOCC/-	[17,181]
122	Machilin D	rat aorta	17.8 µM	d	+						[182]
123	Marrubenol	rat aorta	11.8 ± 0.3 µM ^2^							-VOCC_L_/	[183]
124	Marrubiin	rat aorta	NR	d/i	+					-VOCC/	[184]
125	10-Methoxyaffinisine	rat aorta	NR	d/i	+				x	-VOCC/	[70]
126	Methyl brevifolincarboxylate	rat aorta	NR	i						-ROCC/x	[185]
127	Methyleugenol	rat mesenteric	NR	d/i	+						[67]
128	Methylpaeoniflorin	rat aorta	10.1 µM ^1^	d	+						[186]
129	Milonine	rat mesenteric	1.1 µM ^1^	d/i	+	x			+BK_Ca_, +SK_Ca_,_ +_K_ATP_	- VOCC,- ROCC/ -IP_3_R,-RyR	[187]
130	Mollic acid glucoside	rat aorta	NR	d	+						[188]
131	Morolic acid	rat aorta	94.19 µM ^5^	d	+	x					[189]
132	Moronic acid	rat aorta	16.11 µM ^5^	d	+	x					[189]
133	(+)-Nantenine	rat aorta	NR	i					x	-VOCC/x	[190]
134	(+/−)-Naringenin	rat aortarat aortarat aorta	71.2 ± 5.3 µM ^1^ 4.68 µM ^5^	i i i			-	-	+BK_Ca_	-VDCC, -ROCC/	[17] [18] [19]
135	Naucline	rat aorta	20 µM ^1^	i					x	-VOCC, -ROCC/	[89]
136	1-Nitro-2-phenylethane	rat aorta	231.5 µM ^1^	i	+	x			+K_ATP_, +K_V_		[191]
137	Norathyriol	rat aorta	NR	i	x	x				-VOCC, -ROCC/	[192]
138	Oleanolic acid	rat aorta	5.58 ± 1.28 µM ^1^	d	+	x					[134]
139	12-*O*-Methylcurine	rat aorta	63.2 ± 8.8 µM ^1^	i				-		-VOCC,- ROCC/ -IP_3_R	[193]
140	Orientin	New Zealand rabbit aorta	2.28 µM ^1^	d/i	+	x			x	- VOCC,- ROCC/-	[194]
141	Osthole	rat aorta	NR	i	↑cGMP	x				- VOCC,- ROCC/-	[195]
142	Paeoniflorin	rat aorta	19.4 µM ^1^	d	+						[186]
143	Paeonidanin	rat aorta	7.9 µM ^1^	d	+						[186]
144	Pecrassipine A	rat aorta	NR	d/i	+				x	- VOCC,- ROCC/	[78]
145	1,2,3,4,6-Penta-*O*-galloyl-β-d-glucose	rat aorta	3.6 µM ^1^	d	+	+			x		[196]
146	Perrottetin	rat aorta	NR	i					x	- VOCC,- ROCC/	[56]
147	Phlomeoic acid	rat aorta	NR	d/i	+					-VOCC/	[184]
148	Phloretin	rabbit coronary	NR	i							[149]
149	Piceatannol	rat aorta	2.4 ± 0.4 µM 1	d	+	x			+BK_Ca_		[32]
		rat aorta		d	+						[33]
150	Pimaradienoic acid	rat aorta	NR	i	+	+			x	-VOCC/x	[197]
151	Pinocembrin	rat aorta	4.37 ± 0.02 ^§,5^	d/i	+	x			+K_ATP_, +K_V_	- VOCC/- IP_3_R	[198]
152	Piperitol (sesamin metabolite)	rat aorta	NR	d	+						[119]
153	Plagiochin A	rat aorta	NR	d	+						[56]
154	Polygodial	rabbit pulmonary	NR	d	+	x			x		[37]
		rat portal						-		-VOCC/	[38]
155	Pomolic acid	rat aorta	2.45 μM ^5^	d	+	x			+K_ATP_		[199]
156	(+) Praeruptorin A	rat aorta	35.4 ± 3.6 µM ^1^	d	+				x	- VOCC,- ROCC/ -IP_3_R	[200]
157	(−) Praeruptorin A	rat aorta	45.8 ± 2.5 µM ^1^	i	x				x	-VOCC, -ROCC/ -IP_3_R	[200]
158	Proanthocyanidins*	rat aorta	NR	d	+						[50]
159	Procyanidins*	human internal mammary	NR	d	+	+			+K_ATP_, +SK_Ca_, +K_V_, +K_IR_		[201]
		rat aorta		d	+						[202]
		porcine coronary		+	+						[203]
160	Protosappanin D	rat aortarat mesenteric	NR	d/i	+	+					[85]
161	Puerarin	rat basilar	304 ± 49 µM ^3^	d/i	+	x			+	x/	[115]
162	Quercetin	rat aorta	NR	i	x + + x	+ x	x	-	+BK_Ca_		[24]
		rat coronary	3 mM 7	d/i							[25]
		pig coronary	NR	i							[27]
		rat aorta	4.68 ± 0.08 ^§,5^	i							[163]
		rat mesenteric	5.35 ± 0.15 ^§,5^	i							[163]
		rat aorta	4.36 ± 0.05 ^§,1^	d							[204]
		rat portal	59.5 ± 11.1 µM ^4^	i							[205]
163	Quercetin 3,7-dimethyl ether	rat aorta	4.70 ± 0.18 ^§,1^	d	+						[206]
164	Quercetine-3-*O*-galactoside	rat basilar	20.4 ± 4.49 µM ^3^	d/i	+	+			+		[207]
165	Resveratrol	rat aorta	4.52 ± 0.11 ^§,1^ 4.99 ± 0.11 ^§,1^	i	+				+K_V_ +K_V_	-VOCC/	[35]
		rat aorta		d/i							[208]
		rat mesenteric		d/i							[209]
166	Reticuline	rat aortarat aorta	40 ± 10 µM ^1^ NR	d/i	+	x				- VOCC_L_/- IP_3_R -VOCC_L_/	[63] [210]
167	Rhynchophylline	rat aorta	20–30 µM ^2^	i	x					- VOCC_L_/- IP_3_R,- RyR	[164]
168	Riccardin A	rat aorta	NR	d	+						[56]
169	Riccardin C	rat aorta	NR	d	+						[56]
170	Riccardin F	rat aorta	NR	d	+						[56]
171	Roseoside	rat aorta	NR	d	+						[55]
172	Rotundifolone	rat aorta	184 ± 6 µg/mL ^1^	d/i	+	+			+BK_Ca_	- VOCC_L_/- IP_3_R -VOCC_L_/	[40]
		rat aorta	NR	i							[41]
		rat mesenteric	4.0 ± 0.02 ^§,1^	d/i							[42]
173	Rutaecarpine	rat aorta	NR	d	+				x	-/- - VOCC_L_/- IP_3_R	[43]
		rat aorta		d	+						[44]
		rat aorta									[45]
174	Rutin	rat mesenteric rat aorta	NR	d	+	+			+K_ATP_		[211]
175	Salvianolic acid B	rat coronary	147.9 ± 17.4 µg/mL ^6^	i					+	-VOCC/	[212]
176	Sanguinarine	rat aorta	3.18 ± 0.37 µM ^1^	i						-VOCC, -ROCC/ -IP_3_R	[213]
177	Saponins from Ginseng*		NR							-ROCC/	[49]
178	Sappanchalcone	rat aortarat mesenteric	NR	d	+	+					[85]
179	Saucerneol	rat aorta	2.2 µM	d	+						[182]
180	Saucerneol D	rat aorta	12.7 µM	d	+						[182]
181	Scirpusin B	rat aorta	NR	d	+						[214]
182	Scutellarin	rat aorta	7.7 ± 0.6 µM ^5^	i	x	x		x	x	-VOCC/x	[215]
183	Senkyunolide A	rat aorta	4.32 ± 0.10 §,^1^	i	x	x			x		[180]
184	S-petasin	rat mesenteric	6.01 ± 0.08 ^§,3^	i i	x x	x x				- VOCC_L_/ - VOCC_L_/	[72]
		rat aorta	4.76 ± 0.16 ^§,3^								[72]
		rat aorta	6.6 ± 1.4 µM ^2^								[216]
185	Tetramethylpyrazine	rat aorta	NR	d/i	+ +				+K_ATP_, +SK_Ca_	-VOCC/	[217]
		rabbit basilar	NR								[218]
		rat aorta	NR								[219]
		rat pulmonary	522 µM ^1^								[220]
186	Tetrandrine		NR							-VOCCL/	[217]
187	Thaligrisine	rat aorta	23.0 ± 0.39 µM ^5^							-VOCC/	[221]
188	Thymol	rat aorta	106.4 ± 11.3 µM ^1^	i	x			-		-VOCC/-IP3R	[96]
189	Tilianin	rat aorta	240 µM ^5^	d/i	+	x			+ K_V_		[222]
190	Trans-dehydrocrotonin	rat aorta	NR	d	+						[223]
191	Trans-resveratrol	rat aorta	3.12 ± 0.26 µM ^1^	d	+						[224,225]
192	Ursolic acid	rat aorta	44.1 ± 6.1 µM ^5^	d	+	x					[64]
193	Villocarine A	rat aorta	NR	d/i	+				+	-VOCC, -ROCC/	[226]
194	Vincamedine	rat aorta	NR	d/i	+				x	-VOCC, -ROCC/	[227]
195	Visnadine	rat aortarat portal	NR					-		-VOCC_L_/	[228]
196	Visnagin	rat aorta	22 ± 4 µM 5	i				-		-VOCC_L_, -ROCC/	[229]
										-IP_3_R,-RyR	
197	Vitisin C	rabbit aorta	NR	d	+						[230]
198	Vulgarenol	guinea pig heart	NR	d	+						[231]
199	Wine polyphenolic compounds *	rat aorta	3.27 ± 0.02 ^§,5^	d	+			x	+		[47,178]
200	Xanthoangelol	rat aorta	NR	d	+					-VOCC/	[53]
201	Xanthoangelol B	rat aorta	NR	i	x					-VOCC/	[53]
202	Xanthoangelol E	rat aorta	NR	d	+					-VOCC/	[53]
203	Xanthoangelol F	rat aorta	NR	d	+					-VOCC/	[53]
204	Xanthone	rat aorta	60.26 ± 8.43 µM 5	i		↑cAMP				-VOCC, -ROCC/x	[232]
205	Xanthorrhizol	rat aorta	NR	i	x	x				-VOCC, -ROCC/	[233]
206	Zearalanone	rabbit coronary	NR	i						-VOCC/	[149]
207	(*Z*)-3-methylthioacrylic ester of 2beta-hydroxy-8betaH-7(11)-eremophilene-12,8-olide	rat mesentericrat aorta	5.24 ± 0.13 ^§,3^ 4.26 ± 0.17 ^§,3^	i	x	x				-VOCC_L_/	[72]

*Abbreviations:* d, endothelium-dependent; i, endothelium-independent; +, activation; -, inactivation; x, without involvement; EC_50_, median effective concentration; NO/cGMP, NO/cGMP pathway; PGI_2_/cAMP, PGI_2_/cAMP pathway; PDE, phosphodiesterase; PKC, protein kinase C; Ca^2+^_ext_, extracellular Ca^2+^ influx; Ca^2+^_int_, Ca^2+^ release from intracellular stores; ↑cGMP, increased levels of cGMP; ↑cAMP, increased levels of cAMP; BK_Ca_, high-conductance Ca^2+^ activated K^+^ channels; IK_Ca_, intermediate-conductance Ca^2+^-activated K^+^ channels; SK_Ca_, low-conductance Ca^2+^-activated K^+^ channels; K_ATP_, ATP-dependent K^+^ channels; K_IR_, inwardly rectifying K^+^ channels; K_V_, voltage-dependent K^+^ channels; VOCC, voltage-operated Ca^2+^ channels, VOCC_L_, L-type voltage-operated Ca^2+^ channels; ROCC, receptor-operated Ca^2+^ channels; IP_3_R, inositol triphosphate receptor; RyR, caffeine/ryanodine receptor. EC_50_ determined in tissues precontracted with ^1^ phenylephrine, ^2^ KCl, ^3^ U46619, ^4^ prostaglandin F2α, ^5^ norepinephrine, ^6^ 5-hydroxytryptamine, ^7^ 4-aminopyridine. § pD2 (−log EC_50_); ǂ pIC30 (−log IC_30_). NR, not reported; No symbol, not investigated; * Mixtures of compounds obtained from a single plant species.

## 6. Compounds Acting on the NO/cGMP Pathway

Although three distinct isoforms of NO synthase (NOS) have been identified (endothelial, eNOS; inducible, iNOS; and neuronal, nNOS), it has generally been accepted that regulation of vascular tone is primarily dependent upon the release of NO from eNOS [234]. However, some studies have suggested that nNOS [235] and iNOS [236] may also be involved in this process. Therefore, NO synthesis can be modulated by regulating the activity or gene expression of the three NOS isoforms [237]. NO, produced by these enzymes, dilates all types of blood vessels by stimulating sGC and increasing cGMP in smooth muscle cells [238].

### 6.1. Compounds that Regulate eNOS Expression

Although eNOS was initially characterized as a constitutive enzyme of the vascular endothelium, there is evidence to suggest that the expression of this enzyme can be regulated by physiological stimuli or by the actions of certain compounds [239,240]. Some of the compounds obtained from plants that regulate the gene expression of eNOS are betulinic acid, a pentacyclic triterpene isolated from *Zizyphi spinosi*, a plant used in traditional Chinese medicine for the treatment of cardiovascular diseases [241]; several flavonoids, such as cynaroside and luteolin, which are constituents of the plants *Cynara scolymus* L. (artichoke) and *Prunella vulgaris* [242,243]; alkaloids, such as keayanidine B and keayanine, isolated from *Microdesmis keayana*, an African tropical plant whose roots are used in traditional medicine for treating erectile dysfunction [172]; and other metabolites, such as piceatannol [244].

In general, assays for determining the contributions of these compounds to the regulation of eNOS gene expression have been performed on endothelial cells from the human umbilical cord vein (the EA. hy926 cell line) [244]. For example, in the study of icariin, a flavonoid isolated from *Epimedii herba*, this cell line was cultured in the presence of different concentrations of it. Subsequently, reverse transcriptase PCR and western blot techniques were used to determine the change in the levels of mRNA and protein of eNOS, respectively. The results indicated that after incubation for 12 h in the presence of icariin, both the mRNA expression and the protein levels of eNOS increased significantly as a function of time and concentration. Additionally, icariin induced a significant relaxation on rat aorta and canine coronary artery [158,159]. 

### 6.2. Compounds that Regulate eNOS Activity

In general, assessment of the participation of the NO/cGMP pathway is accomplished through the use of inhibitors of eNOS and sGC. In the case of eNOS, the most commonly inhibitor used is N_ω_-nitro-L-arginine methyl ester (L-NAME) or some other derivatives, such as N^G^-monomethyl-L-arginine (L-NMMA) [82,162]. In the case of sGC, 1*H*-[1,2,4]oxadiazole[4,3-a]quinoxaline-1-one (ODQ) or methylene blue [125] are the most commonly used inhibitors. 

The tissues commonly used to test the effects of compounds on the NO/cGMP pathway are isolated rat thoracic aorta rings or arteries from the mesenteric artery bed [126,187]. However, other tissues have been used, such as rat basilar artery [115], rabbit thoracic aorta [230], porcine coronary artery [113], canine coronary artery [159], and bovine ophthalmic artery [31]. An example of a study where both models, the isolated aorta and the mesenteric artery bed, were employed comprises evaluation of the vasodilator effect of alpha-terpineol and methyl eugenol, which were obtained from the essential oil of *Croton nepetaefolius*. It was found that the NO/cGMP pathway was involved in the vasodilatory activity of these compounds, as the pathway was inhibited in the presence of L-NAME and methylene blue [67].

An example of a compound whose mechanism of action involves activation of eNOS is brazilin, a homoisoflavonoid obtained from *Caesalpinia sappan L*. This metabolite induced an increase in cGMP levels and vasodilation of the aorta in a concentration-dependent manner. The effect of brazilin has also been studied in cultured endothelial cells from the umbilical cord vein. In these cell cultures, brazilin induced a concentration-dependent increase in eNOS activity by causing an elevation of intracellular Ca^2+^ in endothelial cells, thus stimulating calmodulin, which in turn activated eNOS [84]. A similar mechanism of action was proposed for gomisin A, a lignane obtained from *Schisandra chinensis;* however, in this case, human coronary endothelial cells were used to determine the activation of eNOS [245].

Mechanisms that activate eNOS through the phosphatidylinositol-3-kinase/protein kinase B (PIK3/Akt) pathway have also been proposed. The vasodilator effect of epigallocatechin-3-gallate, the most abundant catechin in tea (*Camellia sinensis*), was dramatically reduced by the PIK3 inhibitor wortmannin and the Akt inhibitor SH6, suggesting that this compound activates the NO/cGMP pathway by inducing the phosphorylation of eNOS [31]. Moreover, this mechanism has also been suggested to account for the vasodilatory activity of proanthocyanidins from the persimmon leaf, quercetin and resveratrol. The effect of these metabolites was studied in diverse cultured endothelial cells and results have pointed out that these compounds induced vasorelaxation through the endothelium-dependent NO/cGMP pathway via sequential phosphorylation of Akt [28,36,50].

### 6.3. Compounds that Regulate the Activity and Expression of sGC

The results of some studies have suggested that the vasodilator effects of certain compounds produced from plants are mediated by the activation of sGC and, therefore, by an increase in cGMP levels. The levels of sGC have been quantified on rings of isolated rat aortas using immunological techniques [45,162]. In this context, it has been proposed that isoliquiritigenin, a chalcone isolated from *Dalbergia odorifera*, relaxes the aorta by an endothelium-independent mechanism. Furthermore, incubation of the aorta with this chalcone caused an increase in cGMP levels and a slight increase in cAMP [162]. It has also been proposed that the metabolites emodin and osthole produce their vasodilator effects through a mechanism of action involving increased levels of sGC [127,195]. 

About 40% of the compounds showed more than one mechanism of action (Table 1). For example, alpinetin and cardamonin exert their relaxing effects through both endothelium-dependent and endothelium-independent mechanisms, the former by activation of the NO/cGMP pathway and the latter through the non-selective inhibition of Ca^2+^ channels in smooth muscle cells and the inhibition of the contractile mechanism dependent on protein kinase C (PKC) [69]. Similar mechanisms have been proposed for citral and formononetin; both compounds induced relaxation in rat aortic rings through an endothelium-dependent manner via the nitric oxide pathway, and also involving endothelium-independent vasodilatation by the blockade of Ca^2+^ channels [102,145]. 

It has also been suggested that the involvement of different mechanisms could depend on the concentration of the metabolite. Low concentrations of caffeic acid phenylethyl ester (CAPE), one of the main components of propolis, induce a relaxing effect on vascular smooth muscle through the activation of the NO/cGMP pathway. In contrast, high concentrations of this compound induce vasodilation in an endothelium-independent manner, likely due to the inhibition of Ca^2+^ entry into the cytoplasm of muscle cells or due to the inhibition of the release of this cation from intracellular stores [91]. 

Moreover, the mechanism of action depends on the type of vascular bed and species variations. In this sense it has been demonstrated that vascular relaxation attributable to NO is most prominent in large vessels such as the aorta, while in resistance vessels that regulate blood pressure more directly, NO’s effects are less evident [246]. As an example of the influence of species variations on the action of compounds that affect NO expression, it was shown that resveratrol induced down-regulation of eNOS gene expression in human endothelial cells [247], in contrast, this compound increased eNOS protein expression in bovine endothelial cells [248]. On the other hand, imperatorin, a coumarin obtained from *Angelica dahurica* var. formosana, induced an endothelium-independent relaxation in rat mesenteric arterial rings by blocking the voltage-dependent calcium channel and the receptor-mediated Ca^2+^ influx and Ca^2+^ release [160]. However, in mouse thoracic aorta this coumarin elicited vasodilatation via an endothelium-dependent mechanism involving the nitric oxide pathway [161].

Some studies have conducted *in vivo* assays in addition to tests on isolated tissues. Chrysin glucoside, isolated from the leaves and flowers of *Calycotome villosa*, has been observed to have an endothelium-dependent vasodilator effect on isolated rat aortas and a hypotensive effect when administered intravenously to rats [249]. The results of the *in vivo* assays suggest that the hypotensive effect is probably due to increased vascular relaxation [22,63,76,107,119,136,165].

## 7. Compounds that Activate the PGI2/cAMP Pathway

Few studies have proposed the activation of the PGI_2_/cAMP pathway as a mechanism for the vasodilator effects of plant-derived compounds. PGI_2_ is an endogenous vasoactive eicosanoid produced by cyclooxygenase (COX) from arachidonic acid in endothelial cells; its production is stimulated by endogenous agonists such as serotonine, histamine, bradykinin and acetylcholine. In addition to inhibiting platelet aggregation, PGI_2_ also causes relaxation of vascular smooth muscle through stimulation of a G-protein-coupled receptor that, in turn, activates adenylyl cyclase (AC) and thus raises cAMP levels, inducing vasodilation as a result [250]. The participation of this pathway is determined by using indomethacin as an inhibitor of the COX enzyme [82,154]. Some compounds whose mechanism of action involves the activation of this pathway at the level of the endothelium are ethyl cinnamate, isolated from the rhizomes of *Kaempferia galanga* [104]; eudesmin, a lignan obtained from *Piper truncatum* [135]; labdane-302, a diterpene obtained from *Xylopia langsdorffiana* [174]; rutin [211]; and procyanidins, derived from grape seeds [201].

The vasodilator activity of procyanidins was evaluated in human internal mammary aortic rings. It was determined that both the NO/cGMP and the PGI_2_/cAMP pathways were involved in this process through experiments using inhibitors of eNOS (L-NMMA) and sGC (ODQ) for the first pathway and COX (indomethacin) for the second one. The vasodilator effect of procyanidins was eliminated by the removal of the endothelium. Additionally, inhibition of COX produced a 50% decrease in the vasodilatory activity of these compounds, suggesting the involvement of the PGI_2_/cAMP pathway in their mechanism of action. Subsequent experiments confirmed this finding by observing an increase in PGI_2_ release, which was dependent on the concentration of procyanidins [201]. 

Other studies have suggested that some natural compounds produce a vasodilator effect by directly activating AC or increasing cAMP levels in smooth muscle cells. The experimental protocols of these studies aimed to evaluate the effects of both an AC inhibitor (SQ22536) and an inhibitor of cAMP-dependent protein kinase (PKA) (KT5720) on the vasodilation produced by the test compound [79]. Additionally, analogs and antagonists of cyclic nucleotides have been used in the evaluation of these pathways [251]. For example, puerarin, an isoflavone isolated from *Radix puerariae* that was evaluated using porcine coronary artery rings, was able to shift the dose-response curve of sodium nitroprusside (SNP) to the left. This effect was independent of the endothelium. The SNP-induced relaxation was enhanced by the cAMP analog, 8-Br-cAMP, at a rate similar to that of puerarin, suggesting the involvement of the PGI_2_/cAMP pathway in the increased vasodilatory activity. Moreover, the cAMP antagonist Rp-8-Br-cAMP decreased the vasoactive effect of this isoflavone. In this case, analogs of cGMP (agonists or antagonists) had no effect on the activity of puerarin. Based on these results, it was suggested that the mechanism of action whereby this isoflavone increases vasodilation in the porcine coronary artery is the activation of the PGI_2_/cAMP pathway [251].

## 8. Compounds that Inhibit Phosphodiesterases (PDEs)

Cyclic nucleotide phospodiesterases (PDEs) are enzymes that regulate the cellular levels of cAMP and cGMP by controlling their rates of degradation [252]. The major PDEs in arterial smooth muscle are PDE1, PDE3, PDE4 and PDE5; specifically, PDE5 has been found to be a major cGMP-hydrolizing PDE expressed in smooth muscle cells. The inhibition of PDEs produces vasorelaxant effects by increasing cyclic nucleotide levels [252,253,254].

Several compounds, mostly flavonoids, have been described as PDE inhibitors and vasodilators [18,23,29,157]. The involvement of PDEs in the vasorelaxant effect of these compounds was evaluated by measuring the change on PDE activity. PDEs have been isolated from the cytosolic fraction of bovine aortic smooth muscle [18,23] or rat aorta [87] and their activities were measured by radioenzimatic assays [255]. 

Specific PDEs were inhibited by different compounds. For example, the vasorelaxant effect of dioclein inhibited PDE1, and to a lesser extent PDE4 and PDE5 [23]; meanwhile, epigallocatechin-3-gallate showed activity over PDE1 and PDE2 [29], while butein, a chalcone obtained from *Dalbergia odorifera*, inhibited PDE4 only [87].

## 9. Compounds that Activate K+ Channels

The K^+^ channels in vascular smooth muscle play an important role in vasodilation because the outflow of K^+^ through these channels hyperpolarizes the membrane and thereby inhibits the entry of Ca^2+^. This process eventually results in the relaxation of blood vessels [256]. Four different types of potassium channels have been characterized in arterial smooth muscle: voltage-dependent channels (K_V_), Ca^2+^-activated channels (large-conductance, BK_Ca_; intermediate-conductance, IK_Ca_; and small-conductance, SK_Ca_), ATP-dependent channels (K_ATP_) and inwardly rectifying channels (K_IR_) [257,258,259,260]. It is worth mentioning that there is evidence for cell to cell, segment to segment, and vascular bed to bed diversity of K^+^ channels that could explain the varying responses of arterial segments or different arteries to stimuli such as hypoxia, vasoactive drugs, or arterial wall injury [261,262,263]. 

The involvement of different types of K^+^ channels has been evaluated by the use of channel-specific blockers. The following are the most commonly used blockers of K^+^ channels: chloride tetraethylammonium (TEA) and BaCl_2_ as nonselective inhibitors [22,86]; glibenclamide, an inhibitor of K_ATP_ channels; aminopyridine (4-AP), which blocks K_V_ channels; and iberiotoxin [35] and charybdotoxin, which block BK_Ca_ channels [42,98]. In addition, TEA [82], BaCl_2_ [22], and apamin [31] have been used to block BK_Ca_, K_IR_, and SK_Ca_ channels, respectively.

BK_Ca_, highly expressed in vascular smooth muscle cells [258], can be activated by both, the NO/cGMP pathway [264] and EDHF [265]. These channels play a key role in blood pressure regulation and therefore, they have been suggested as novel potential drug targets for the treatment of cardiovascular diseases [266]. Recently, a considerable number of natural compounds, especially of the flavonoid type, have been shown to have a vasodilator effect caused, at least in part, by activation of BK_Ca_ channels [19,22,198,267,268]. Other compounds with different chemical structures that activate this kind of potassium channels are: diosgenin (steroid sapogenin) [62]; piceatannol (stilbene) [32], isolated from the root of *Rheum undulatum*; and rotundifolone (monoterpene) [42], the major constituent of the essential oil of *Mentha x villosa* Hudson. 

The study of compounds that activate K^+^ channels also includes the use of electrophysiological techniques, both to demonstrate these compounds’ role as stimulants and to characterize the type of channels involved in their vasodilator mechanisms. The most common strategy is the patch-clamp technique used on isolated muscle cells [116] or in *Xenopus* oocytes that express K^+^ channels from other organisms [269]. For example, the elucidation of the mechanism of action of rotundifolone was carried out in rat superior mesenteric arteries. For investigating the involvement of K^+^ channels in the vasorelaxant mechanism, several specific channel blockers were used such as TEA, charybdotoxin, 4-AP and glibenclamide. In addition, electrophysiological testing using the patch-clamp technique in mesenteric smooth muscle cells was used to identify the channels activated by rotundifolone. The results indicated that the vasodilator effect of this compound involves the participation of BK_Ca_ channels [42]. However, it has been shown that the use of the patch-clamp technique induces apparent phenotypic changes, particularly when it is used on isolated and cultured cells, compared to data derived from intact tissue. Consequently, data gathered in this manner should be interpreted with caution [270].

## 10. Compounds that Decrease Intracellular Ca^2+^ Concentration

The mechanism of vascular smooth muscle contraction involves the participation of different signal transduction pathways, all of which converge to increase cytoplasmic Ca^2+^ concentrations. The concentration of this cation increases both by extracellular Ca^2+^ entering through voltage-operated Ca^2+^ channels (VOCCs) and receptor-operated Ca^2+^ channels (ROCCs), and by the release of Ca^2+^ from intracellular stores [123]. Therefore, the mechanisms of action associated with vasodilating agents that decrease intracellular Ca^2+^ concentration involve blocking VOCCs and ROCCs or inhibiting the release of this cation from intracellular stores. The experimental strategy to determine the involvement of Ca^2+^ channels in the vasodilating effect of test compounds involves incubating aortic rings in a Ca^2+^-free medium containing a high concentration of K^+^ and to which CaCl_2_ is gradually added to induce contraction, both in the absence and presence of the vasodilating compound [79,123]. 

Different techniques are used to determine the involvement of VOCCs, ROCCs or the release of intracellular calcium. The inhibitory action of vasodilator compounds on VOCCs can be seen as a rightward shift in the dose-response curve for CaCl_2_, as noted in the case of ligustilide, a compound extracted from *Ligusticum chuanxiong*, a plant used in traditional Chinese medicine [179], and naucline, an alkaloid derived from *Nauclea officinalis* [89]. For evaluating the involvement of ROCCs, dose-response curves are performed in the presence of an adrenergic agonist, such as noradrenaline (NA) [123] or phenylephrine (PE) [56] to induce contractions, both in the absence and the presence of the vasodilator compound [89,123]. In addition, the contribution of Ca^2+^ released from intracellular stores is determined by incubating the tissue in a Krebs solution free of Ca^2+^ and to which NA is subsequently added to induce phasic contractions with calcium from the sarcoplasmic reticulum. Subsequently, once the contraction is stabilized, CaCl_2_ is added to induce a tonic contraction. When incubating segments of the aorta with the test compound under these conditions, a decrease of phasic contractions signals that the effect is produced by the outflow of intracellular Ca^2+^, whereas a decrease in tonic contraction signals that the effect is mediated by Ca^2+^ entry through ROCCs [185]. 

The release of Ca^2+^ from intracellular stores is regulated by the inositol-1,4,5-triphosphate (IP_3_) system and by the ryanodine receptors (RyRs). RyRs system are a Ca^2+^ release system where Ca^2+^ release is induced by the presence of Ca^2+^ when the receptors are activated by caffeine [179]. For example, isopropyl-3-(3,4-dihydroxyphenyl)-2-hydroxypropanoate has been shown to inhibit both KCl-induced and norepinephrine-induced contractions in the absence and presence of Ca^2+^ in the rat mesenteric artery. These results suggest that in addition to its activity on VOCCs, this compound also acts on ROCCs and on intracellular calcium stores [123]. In this type of study, blockers of L-type Ca^2+^ channels, such as nifedipine [271] or diltiazem [154], are used as a positive control. However, calycosin, the main component of *Astragali radix*, was shown to inhibit CaCl_2_-induced vasoconstriction in the presence of KCl and PE but did not affect PE-induced contractions in a calcium-free medium. These results indicated the involvement of VOCCs and ROCCs in the vasodilator effect produced by calicosin, excluding the outflow of intracellular Ca^2+^ [93]. In contrast, low concentrations of euxanthone, a metabolite isolated from *Polygala caudate*, inhibited the phasic contraction, suggesting that the exit of Ca^2+^ from the endoplasmic reticulum is involved in the relaxing activity [139]. Moreover, both cardamonin and alpinetin can inhibit the transient contractions produced by PE and caffeine in a Ca^2+^-free medium and also the contractions induced by K^+^. The authors suggest that these compounds act through the nonspecific inhibition of Ca^2+^ entry and the release of intracellular Ca^2+^ [69]. 

Other methodologies have been used to elucidate the mechanisms of action of vasoactive compounds. For example, the involvement of VOCCs in the vasodilator mechanism of marrubenol, a diterpene extracted from *Marrubium vulgare*, was confirmed by recording the inflow current through calcium channels using patch-clamp and fluorescence techniques [183]. 

## 11. Compounds that Activate Endothelial Transient Receptor Potential (TRP) Cation Channels

Transient receptor potential (TRP) cation channels are currently considered as the leading candidate proteins mediating diverse non-voltage-gated calcium entry pathways in vascular endothelium and smooth muscle [272,273]. The TRP superfamily contains three major subfamilies based on sequence homology: TRPV (vanilloid), TRPC (canonical), and TRPM (melastatin). Moreover, three additional subfamilies (the “distant TRPs”), TRPP (polycystin), TRPML (mucolipin), and TRPA (ankyrin) have been proposed [274]. In particular, the endothelial TRP channels are exposed to different agonists that enter the blood stream as dietary molecules. Some of these molecules, found in commonly consumed food and plants used in traditional medical practices of several cultures are able to activate these kinds of channels [97,272,273]. Carvacrol, one of the major components of oregano (*Origanum vulgare*) essential oil, induces an endothelium-dependent vasodilation by activating TRPV3 [97]. Recently, it has been reported that allyl isothiocyanate, which is found in the seeds of mustard (*Brassica nigra* and *B. juncea*) causes endothelium-dependent vasodilation of rat cerebral arteries by a mechanism involving TRPA1 activation [66]. 

## 12. Compounds that Inhibit Protein Kinase C

The mechanism of vascular smooth muscle contraction evokes the phosphorylation of myosin light chain by increasing intracellular Ca^2+^ concentration. Additionally, the decrease of the myosin light chain phosphatase (MLCP) increases the sensitivity to Ca^2+^ [275]. Several pathways have been suggested for the Ca^2+^ sensing mechanism. One of them is the PKC/CPI-17 pathway [276]. PKC phosporilates CPI-17, enhancing its inhibitory activity over MLCP [276] and producing a sustained contraction. PKC has been found in high concentrations in vascular smooth muscle and can be activated by diacylglicerol [277].

Only a few compounds have been found to evoke their vasorelaxant activity through this mechanism; in all cases, PKC inhibition was not the only mechanism. The participation of PKC in the vasorelaxant mechanism has been evaluated using activators of PKC in smooth muscle cells, such as phorbol esters. 12-*O*-tetradecanoyl phorbol 13-acetate, phorbol 12-myristate-13-acetate (PMA) and phorbol 12,13-dibutyrate (PDB) were used to evaluate the vasorelaxant mechanisms for dioclein [21], quercetin [24] and euxanthone [139], respectively. This last activator was used also in the characterization of the mechanism of action for thymol and carvacrol: PDB induced a sustained contraction that was attenuated when thymol or carvacrol were added (300 and 1,000 µM) [96].

## 13. Conclusions

The present review focused on the mechanisms of action responsible for the vasodilator activity of plant-derived compounds. From the information obtained, we identified the main mechanisms of action of most of the vasodilator compounds; these mechanisms are the activation of the NO/cGMP and PGI_2_/cAMP pathways, the activation of K^+^ channels and the blockade of voltage-dependent Ca^2+^ channels. 

It should be noted that more than one mechanism of action has been proposed to be involved in the vasodilator effect of almost half of all of the analyzed compounds. This finding suggests that compounds derived from plants may have great therapeutic potential as they involve multiple mechanisms of action in their vascular relaxing activity. In this context, it is critical to emphasize the importance of understanding the different mechanisms of action in order to establish new therapeutic strategies for addressing various cardiovascular diseases. 

Finally, given the structural diversity of the active compounds derived from natural products and the diversity of mechanisms of action responsible for their vasodilator activity, it is important to continue the search for new active substances that help in the treatment of cardiovascular diseases.
